# Stroke-induced immunosuppression: implications for the prevention and prediction of post-stroke infections

**DOI:** 10.1186/s12974-021-02177-0

**Published:** 2021-06-06

**Authors:** Júlia Faura, Alejandro Bustamante, Francesc Miró-Mur, Joan Montaner

**Affiliations:** 1 Neurovascular Research Laboratory, Vall d’Hebron Research Institute (VHIR), Universitat Autònoma de Barcelona, Barcelona, Spain; 2 Stroke Unit, Hospital Universitari Germans Trias i Pujol, Carretera de Canyet, s/n, 08916 Badalona, Barcelona, Spain; 3 Systemic Autoimmune Research Unit, Vall d’Hebron Research Institute (VHIR), Barcelona, Spain; 4 Stroke Research Program, Institute of Biomedicine of Seville, IBiS/Hospital Universitario Virgen del Rocío/CSIC/University of Seville & Department of Neurology, Hospital Universitario Virgen de la Macarena, Seville, Spain

**Keywords:** Stroke, Immunosuppression, Biomarkers, Inflammation, Infection, Pneumonia

## Abstract

Stroke produces a powerful inflammatory cascade in the brain, but also a suppression of the peripheral immune system, which is also called stroke-induced immunosuppression (SIIS). The main processes that lead to SIIS are a shift from a lymphocyte phenotype T-helper (Th) 1 to a Th2 phenotype, a decrease of the lymphocyte counts and NK cells in the blood and spleen, and an impairment of the defense mechanisms of neutrophils and monocytes. The direct clinical consequence of SIIS in stroke patients is an increased susceptibility to stroke-associated infections, which is enhanced by clinical factors like dysphagia. Among these infections, stroke-associated pneumonia (SAP) is the one that accounts for the highest impact on stroke outcome, so research is focused on its early diagnosis and prevention. Biomarkers indicating modifications in SIIS pathways could have an important role in the early prediction of SAP, but currently, there are no individual biomarkers or panels of biomarkers that are accurate enough to be translated to clinical practice. Similarly, there is still no efficient therapy to prevent the onset of SAP, and clinical trials testing prophylactic antibiotic treatment and β-blockers have failed. However, local immunomodulation could open up a new research opportunity to find a preventive therapy for SAP. Recent studies have focused on the pulmonary immune changes that could be caused by stroke similarly to other acquired brain injuries. Some of the traits observed in animal models of stroke include lung edema and inflammation, as well as inflammation of the bronchoalveolar lavage fluid.

**Supplementary Information:**

The online version contains supplementary material available at 10.1186/s12974-021-02177-0.

## Background

Stroke-induced immunosuppression (SIIS) is a set of processes that lead to a peripheral suppression of the immune system after the occurrence of stroke. One of the main and direct consequences of this SIIS is that it makes stroke patients more susceptible to bacterial infections. Stroke-associated infections (SAIs) represent one of the major complications post-stroke, which worsens the functional outcome of patients and increases their mortality rates [1]. Approximately 30% (24–36%) of patients develop infections after stroke, with pneumonia and urinary tract infections (UTI) being the most common forms, both having a frequency of 10% [2].

Among these infections, stroke-associated pneumonia (SAP) is usually the most acute type of SAI and has the worst impact on functional outcome [3]. It increases mortality for up to 1 year, prolongs hospital stays, and worsens the functional outcome at discharge [4]. The clinical definition of pneumonia after stroke has differed in many studies in both the terminology and the diagnosis of the complication [5]. To address this issue, the Pneumonia in Stroke Consensus (PISCES) Group proposed the term SAP, to encompass all terms referring to lower respiratory tract infections in stroke patients within the first 7 days after stroke onset [6]. In the same publication, new diagnostic criteria were proposed based on the criteria for the definition of healthcare-associated infection from the Centers for Disease Control and Prevention (CDC) of the United States of America, which were the most used criteria by that point [7]. However, SAP assessment is still challenging, especially due to the limited role of chest radiography. For this, chest computed tomography has been proposed as a complement to PISCES criteria in the screening of SAP in stroke patients. In a small cohort, thorax high-resolution computed tomography (THRCT) was able to differentiate between bronchopneumonia and other low respiratory tract infections in SAP patients, demonstrating a high accuracy in the diagnosis of SAP [8]. More recently, Kishore et al. [9] have also addressed this issue, but they have observed that conventional chest X-rays have limited accuracy for the diagnostic of SAP when compared with THRCT. Similarly, they found in their work some discrepancies between PISCES criteria and THRCT. Larger studies, including perhaps serial imaging, are needed in this field, to validate whether PISCES criteria and THRCT might be combined for the diagnosis of SAP.

Nowadays, the clinical strategies against SAP are based on wide-spectrum antibiotics once an infection is diagnosed through clinical criteria, along with prevention by dysphagia screening in stroke units. Recently, the PISCES consortia launched a recommendation for a standardized approach to antibiotic therapy in post-stroke pneumonia [10]. Nonetheless, early treatment before the development of clinical signs could prevent the onset of SAP or ameliorate its consequences. This would have benefits for patients and saving also a great number of resources for health care systems [11]. Several clinical trials have explored this idea through the administration of prophylactic antibiotics to acute stroke patients. However, the meta-analysis performed by Vermeij et al. [12] showed that preventive antibiotics neither reduce the risk of pneumonia nor the risk of death or poor outcome after stroke. The treatment did reduce the occurrence of overall infections and UTIs, but did not show any effect regarding the occurrence of SAP. These results were translated into recommendations against the routine use of prophylactic antibiotics in stroke guidelines.

In addition, antibiotics can have both protective and detrimental effects on the central nervous system (CNS) and could have modulatory effects on the immune system. The administration of antibiotics affects human bacterial communities and may lead to a dysbiosis of intestinal microbiota, which could remain altered for 6 months after antibiotic therapy [13]. Studies in mice living in germ-free conditions showed the importance of gut microbiota in the development and function of the CNS. These germ-free mice developed brain diseases that were replicated in antibiotic-depleted microbiota animals [14]. In experimental stroke, it has been observed that the depletion of gut microbiota by antibiotics worsens the outcome of the ischemic mice, decreasing their survival, and developing acute colitis [15]. However, Benakis et al. found that antibiotic-induced alterations in the gut microbiota decreased ischemic injury in the brain. Concretely, dysbiosis reduced the trafficking of effector T cells from the gut to leptomeninges after stroke. In the brain, these cells enhance neuroinflammation [16]. Further studies are needed to elucidate the effects and interaction of antibiotics with stroke, to determine the impact of the antibiotic treatment in the brain lesion in SAI patients.

The failure of these clinical trials could make the stroke units rethink therapeutic strategies for SAP prevention or early treatment. Diverse reasons might be responsible for the lack of success of these trials, such as the inclusion of patients with a relatively low risk for infection, the wrong choice of the antibiotic regime, or the time-window for patient inclusion. But another plausible explanation is that despite being useful for SAP treatment, antibiotics might not be effective in the prevention of SAP, thus opening a new opportunity for alternative preventive measures, with SIIS as an alternative therapeutic target.

Along these lines, immunomodulation of the peripheral immune system has also been explored as an alternative therapy for the prevention of these infections, but to a lesser extent than preventive antibiotics and mostly in experimental stroke. Developing new immunomodulating preventive therapies against the development of SAP will require a deep knowledge of the mechanisms that lead to SIIS and the resulting susceptibility of stroke patients to bacterial infections.

Hence, this review intends to present those mechanisms implicated in SIIS, focusing on which clinical implications these pathways could have, as the finding of biomarkers capable of predicting the onset of SAI and SAP and the use of immunomodulatory therapies (either systemic or local) to prevent them.

## Cellular and molecular mechanisms of stroke-induced immunosuppression

After the occurrence of stroke, an activation of the inflammatory cascade due to the release of damage-associated molecular patterns (DAMPs) by the injured and dead cells causes a neuroinflammatory state in the brain. The release of inflammatory mediators, together with oxidative stress and other factors, increases the permeability of the brain-blood barrier (BBB), which in turn facilitates leukocyte infiltration into the brain [17].

**Fig. 1:**
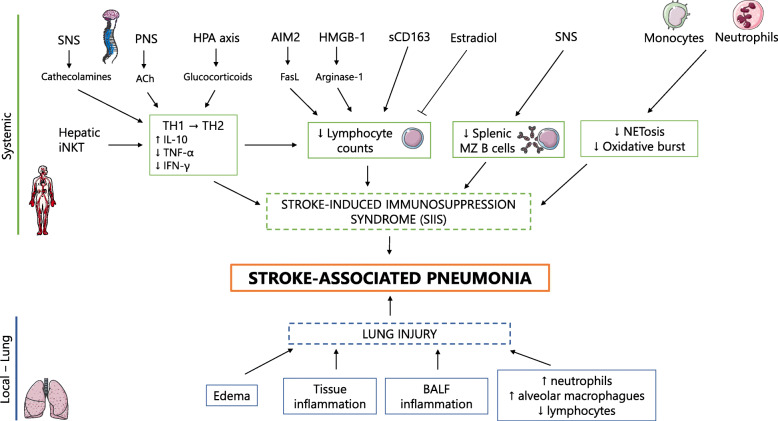
The interplay of local and systemic processes leading to pneumonia in stroke patients. Stroke-associated pneumonia is influenced by systemic and local mechanisms. Locally, there are pulmonary alterations due to stroke itself and the inflammatory processes that develop. BALF inflammation, lung inflammation, and edema seem to be the principal alterations, although there are some discrepancies between studies. On a systemic level, various represented processes lead to 3 main alterations that cause systemic immunosuppression after stroke: an increase of the T-helper (Th) 2/Th1 cytokine ratio; a reduction of the lymphocyte counts in the spleen, thymus, and blood; and a decrease of the antimicrobial defense mechanisms of neutrophils and monocytes. *SNS* sympathetic nervous system, *iNKT* invariant natural killer T cells, *PNS* parasympathetic nervous system, *ACh* acetylcholine, *HPA* hypothalamic–pituitary–adrenal, *HMGB*-1 high motility group box-1, *sCD163* soluble cluster of differentiation 163, *MZ* marginal zone, *FasL* Fas ligand, *BALF* bronchoalveolar lavage fluid. Parts of this figure were supported by Servier Medical Art with permission under the Creative Commons Attribution 3.0 Unported License

Conversely, focusing on the peripheral immune system, immunosuppression has been reported early within the first hours after stroke. One of the main processes of SIIS is the shift of the T cell response from a T-helper (Th) 1 response, characterized by the secretion of pro-inflammatory cytokines such as tumor necrosis factor-α (TNF-α) and interferon-γ (IFN-γ), to a T-helper 2 (Th2) anti-inflammatory response, with the secretion of interleukin (IL)-10 and IL-4 among others. An increased ratio of Th2-type cytokines over Th1-type cytokines characterizes this shift. Another characteristic trait of SIIS is the lymphocyte and splenic impairment, which consists mainly of lymphocytopenia in blood, spleen, and lymph nodes [18]. The molecular and cellular processes that drive to SIIS are described in this chapter, and summarized in Fig. 1.

Various studies have reported a decrease in the production of IFN-γ and the secretion of TNF-α in blood samples from mice whom underwent a middle cerebral artery occlusion (MCAO), as well as an increase of the circulating levels of IL-10 [18, 19]. IL-10 is a well-known anti-inflammatory cytokine that inhibits the production of TNF-α and IFN-γ [20]. A time-dependent increase in IL-10 as well as a decrease in TNF-α/IL-10 ratio was observed in stroke patients developing infection [18]. Jiang et al. [21] observed the same pattern of circulating cytokine expression in human stroke patients. In the same study, they were able to locate this shift in the pattern of circulating cytokine expression, which could occur between days 1 and 3 after stroke.

As mentioned earlier, the shift from Th1 to Th2 is associated with a reduction in lymphocyte counts at the spleen, blood, and thymus. The decrease was observed in MCAO mice 12 h after surgery, and it is due to enhanced apoptosis in these organs [18]. Spleen atrophy characterized by a drastic loss of splenocytes due to apoptosis was observed at 4 days in experimental stroke [22]. In line with this, Yan and Zhang [19] observed a contraction of the spleen volume in MCAO rats beginning at 12 h after the occlusion. This reduction of the splenic volume has also been observed in humans [23, 24].

Although susceptibility to infection after stroke could be eased by SIIS, the latter may represent an evolved mechanism to prevent immune reactions, mainly autoreactive immune responses against CNS molecular components by infiltrating lymphocytes. The efferent neuronal pathways through CNS regulates immune system are presented below.

### SIIS is mediated by the autonomic nervous system

The shift from the Th1 response to the Th2 response seems to be led by the autonomic nervous system (ANS), especially the sympathetic nervous system (SNS). Catecholamines like epinephrine and norepinephrine are SNS mediators that are secreted when the SNS is activated. They act through β-adrenergic receptors of immune cells, suppressing Th1 activities and cellular immune responses through the inhibition of IFN-γ secretion and the stimulation of IL-10 production by immune cells, among other processes [25].

After MCAO surgery in mice, higher levels of metanephrine (MN) and normetanephrine (NMN) (metabolites of epinephrine and norepinephrine, respectively) were observed from 6 h after surgery until 2 weeks. Treating MCAO mice with the beta-blocker propranolol blocks the catecholaminergic receptors and partially inhibits the activation of the SNS in these animals. The effects of this blockade in the circulation were an increase of IFN-γ levels; a decrease of IL-10, MN, and NMN; and an increase of the splenic volume [19]. The inhibition of the SNS through propranolol has also been proven to reduce lymphocyte dysfunction and bacterial infections, as well as improve the survival of MCAO mice [18], thus supporting the relevance of the SNS in SIIS.

β-arrestin2 (ARRB2) has been studied to elucidate the endogenous factors and pathways of the SNS that are involved in SIIS. ARRB2 protein is involved in multiple pathways. Wang et al. [26] found elevated splenic levels of ARRB2 in MCAO rats and a correlation of the protein with sympathetic activity. They also observed dysfunction of splenic monocytes, which was reversed with ARRB2 deficiency in the same manner as propranolol. Hence, these results suggest ARRB2 as an effector of the SNS for the mediation of SIIS.

In fact, ARRB2 seems to be mediating a loss of splenic marginal zone B cells in MCAO animals. McCulloch et al. [27] demonstrated that stroke induces a substantial loss of splenic B cells, especially in those from the marginal B zone. This cell loss is causing impairment to some immune functions, such as those related with the early defense against bacterial microorganisms, and this is associated with spontaneous lung infections. The treatment with propranolol reversed these impairments and reduced the susceptibility to lung infections in MCAO mice.

It is well known that the activity of the hypothalamic-pituitary-adrenal (HPA) axis increases after stroke, with an early increase in the concentrations of cortisol [28, 29]. In a similar manner to catecholamines, glucocorticoids stimulate the secretion of anti-inflammatory cytokines. Prass et al. [18] observed that the inhibition of the HPA axis through the glucocorticoid receptor blocker RU486 prevented lymphopenia, lymphocyte apoptosis, and monocyte dysfunction. However, it did not reduce bacterial infections, unlike propranolol treatment. These results reveal a role of the HPA axis in SIIS, but perhaps not as pivotal as the SNS.

The other division of the ANS, the parasympathetic nervous system (PNS), also seems to play a part in SIIS. The vagus nerve and acetylcholine (ACh) suppress the secretion of proinflammatory cytokines through the binding of acetylcholine to the α7 nicotinic acetylcholine receptor (α7nAChR) in activated macrophages. Specifically, the inhibition of TNF-α production in spleen is driven by ACh-producing T cells [30]. Engel et al. [31] observed a rapid increase of parasympathetic activity in experimental stroke mice through an analysis of heart rate variability and blood pressure. Moreover, MCAO surgery was performed on two types of mice: wild-type (WT) mice who suffered a vagotomy and α7nAChR knockout (KO) mice. Both cases showed a decrease in the bacterial load of the lungs when compared with MCAO WT mice on day 3 after ischemia.

These results suggest that increased parasympathetic activity plays some role in the increased susceptibility to bacterial infections after stroke, possibly through the suppression of innate immune responses of the lungs due to the expression of α7nAChR in activated lung macrophages and alveolar epithelial cells. The results obtained by Lafargue et al. [32] are in line with this hypothesis. They observed attenuation of the effect of stroke in lung injury and mortality when α7nAChR was pharmacologically inhibited or genetically depleted in MCAO mice with pneumonia. Conversely, pretreatment with an α7nAChR activator before MCAO surgery increased lung injury.

### Other immunomediators of SIIS apart from ANS

The Th2/Th1 cytokine ratio is also favored by the hepatic invariant natural killer T (iNKT) cells, producing greater amounts of IL-10, but not IFN-γ. The direct immunomodulation of iNKT cells through the administration of a specific activator (α-galactosylceramide) in MCAO mice reduced stroke-induced neutrophil pulmonary influx, lung edema, and infections. This reduction was observed similarly in MCAO animals treated with propranolol [33].

A decrease in the lymphocyte proliferation also seems to cause the reduction of lymphocyte counts and could be partially explained by a release of arginase I (ArgI) from neutrophils. The enzyme ArgI is constitutively expressed in neutrophil granules, and its main function is to metabolize l-arginine into l-ornithine and urea. Higher levels of this enzyme were found in the blood of MCAO mice on day 4 after ischemia, and the supplementation of l-arginine in vitro in splenic T cells in the same animals restored T cell proliferation and increased IFN-γ production [34]. In humans, *ARG1* mRNA expression and ArgI serum activity correlate with neutrophil-to-lymphocyte ratio (NLR) and stroke severity in stroke patients [35]. These two clinical parameters are associated with SAI [36, 37], thus suggesting a role of ArgI in immune dysfunction.

The release of ArgI could be mediated by high mobility group box 1 (HMGB1), a well-known DAMP, via the receptor of advanced glycation end-products (RAGE). Liesz et al. [38] found that this pathway seems to stimulate the production of myeloid-derived suppressor cells (MDSCs), which could include neutrophils. They also observed that mice with RAGE deficiency after MCAO surgery had reduced splenic lymphocyte apoptosis and increased activated T cell counts. These results suggest that HMGB1 could stimulate the release of ArgI by MDSCs, in turn causing a decrease in leukocyte production.

Soluble CD163 (sCD163) is another molecule that seems to be implicated in the reduction of lymphocyte counts. CD163 is a receptor expressed in the membrane of monocytes and macrophages and is shed through matrix metalloproteinase ADAM17 into sCD163. O’Connell et al. [39] reported higher blood levels of sCD163 and ADAM17 in stroke patients, which negatively correlated with post-stroke lymphocyte counts. In vitro, the authors related the elevations of sCD163 with the suppression of lymphocyte proliferation.

Intense research is being conducted on new signaling pathways implicated in SIIS. Last year, a role of the cluster of differentiation (CD) 200-CD200R1 signaling axis in SIIS was identified. CD200R1 is an inhibitory immune receptor that is expressed in myeloid cells, and it has been observed that CD200R1-KO MCAO mice developed more spontaneous bacterial lung infections than WT MCAO mice, as well as lymphocytopenia and worse functional outcomes, among other effects [40]. These findings suggest that CD200-CD200R1 could be a therapeutic target for immunomodulating preventive therapies of SAI due to its possible protective role against them.

Another mechanism that seems to be implicated in the reduction of lymphocyte counts through the increase of T cell apoptosis is the activation of the AIM2 inflammasome-driven signaling cascade in monocytes [41]. This inflammasome is activated by sensing cell-free DNA, and through the secretion of IL-1β, induces the expression of FasL in monocytes, leading to the T cell apoptosis. Inhibiting this pathway, Roth et al. have observed a reduction of bacterial infections after experimental stroke and an increase of the T cell survival.

### Innate immune cells are also modified after stroke

Apart from lymphocytes, splenic NK cells have also been reported to undergo atrophy and a reduction in the count after stroke, and this process is directly led by SNS and HPA [24]. In the same study, the administration of propranolol and RU486 significantly increased the NK-cell-mediated immune response against post-stroke pneumonia, which improved the mortality rates and reduced the bacterial burden in MCAO mice infected with *Listeria monocytogenes* (LM).

Some functions of the peripheral innate immune cells are altered after stroke, and they could also contribute to SIIS. The mechanisms related to bacterial killing, like oxidative burst and NETosis, are significantly impaired in the monocytes and neutrophils of stroke patients. This impairment was observed upon admission and normalized after day 5 for NETosis. In contrast, the inhibition of the oxidative burst persisted until day 7 [42].

Recently, it has also been described a reduced expression of CD11b in the neutrophils of stroke patients when compared with healthy controls. CD11b recognizes and binds to opsonized bacteria and facilitates the internalization of pathogens, among other functions, and it is considered a leukocyte activation marker [43]. However, in this same study, van Gemmeren et al. did not found an impairment of the neutrophil oxidative burst of stroke patients. The authors ascribe this finding to the small sample size.

Regarding monocytes, Li et al. [44] found overexpression of microRNA-4445 in this leukocyte type. This overexpression seems to be implicated in SIIS, as it causes a suppression of the tumor necrosis factor receptor-associated 4 (TRAF4)/inhibitor of kappa B alpha (IκBα)/nuclear factor kappa B (NF-κB) signaling pathway, activating, in turn, the expression of anti-inflammatory cytokines in monocytes.

### Sex hormones play a part in SIIS

Differences between sexes in stroke have been observed in many studies, and male sex is a risk factor for its incidence and outcome. The difference between sexes seems to diminish with menopause among women, thus suggesting a neuroprotective role of endogenous estrogens against stroke. Male sex has been also described as a risk factor for SAP [45].

For this reason, experimental stroke studies and the study of SIIS are almost always performed with males. However, Zang et al. [46] performed a novel study on SIIS in female mice and described a protective role of estrogens (concretely, estradiol (E2)) in the peripheral immunosuppression of MCAO mice, which improved splenocyte counts. Furthermore, a similar protective role to that of E2 was observed for an agonist of G1: a synthetic agonist of the membrane estrogen receptor G protein-coupled receptor 30. This suggests a potential treatment for SIIS.

Regarding male sexual hormones, Dziennis et al. [47] reported an exacerbation of the peripheral immunosuppression in castrated MCAO mice that were treated with dihydrotestosterone (DHT) replacement. The authors described the role of DHT as a peripheral immunosuppressor following stroke.

Altogether, these antagonistic roles of the sex hormones in immunosuppression could be involved in the fact that male patients have a higher risk of suffering SAP. However, it is also needed to take into account that most women who undergo a stroke are post-menopausal, so these differences among sexes could be lower than expected.

## Clinical implications (1): the use of cellular and molecular changes as biomarkers for the prediction of stroke-associated infections

As mentioned in the “Background” section, there is a lack of preventive therapies for SAI and SAP in clinical practice. The patient selection could be one of the reasons for the failure of the clinical trials with prophylactic antibiotics. Nowadays, apart from dysphagia screening, there are no routine measures to identify patients who have the highest risk of experiencing SAI in clinical practice. The ability to select such patients could represent an improvement in the experimental design of future clinical trials, either with the administration of preventive antibiotics or through immunomodulation. For this reason, research on biomarkers for the prediction of SAI development has been active in the last decade. Different cell and molecular changes related to the mentioned pathways have been explored and could act as predictive biomarkers for SAI or SAP (table S1).

### Cell counts

As a result of leukocyte recruitment to the brain, immunological changes can be observed in circulating immune cells, and systemic inflammation is observed after stroke. In the first hours following stroke, there is an exponential increase in the peripheral neutrophil count and a decrease in the circulating levels of lymphocytes [48]. Due to these changes in peripheral blood cells, different leukocyte counts are being studied as biomarkers in stroke research, such as white blood cell (WBC) count and, recently, the NLR. The leukocyte counts and ratios are easily detectable and they make the early determination a possibility, which means they could potentially be useful biomarkers.

WBC count was explored as a predictor of SAI, SAP, and UTI, and the results indicated it could be an independent predictor of these complications in acute stroke patients [49]. Higher levels of circulating NK cells within the first hours after stroke were observed in patients who developed SAI in a study with 59 participants [50]. NLR is a well-known marker of systemic inflammation. Higher levels of NLR have been found in SAP patients, and there seems to be an association between NLR levels and the severity of SAP [37].

Monocyte to high-density lipoprotein (HDL) cholesterol ratio (MHR) has emerged as a novel inflammation marker. In a recent study, Sun et al. [51] showed the value of MHR and monocyte count as predictors of SAP at admission in a study with more than 800 SAI patients.

### Clinical routine and nutritional-related molecules

Blood biomarkers that are measured routinely in most patients in clinical practice are also good candidates for SAI prediction due to their accessibility, in a similar manner to leukocyte counts. In non-diabetic stroke patients, hyperglycemia upon admission was found to be independently associated with SAI, SAP, and UTI, as well as a poor functional outcome at 3 months after the stroke episode [52]. Rodriguez-Sanz et al. [53] showed an inverse correlation between HDL cholesterol levels and the development of SAI. After establishing a cut-off point for SAI prediction (38.5 mg/dl), higher HDL was significantly associated with a lower risk of developing SAI.

Biomarkers related to nutritional status have also been explored. Serum albumin levels were identified as an independent predictor of SAP in a cohort of 705 patients [54]. Furthermore, lower levels of serum prealbumin were associated with a greater risk of SAI in a smaller cohort of 104 ischemic stroke patients [55]. Huang et al. [56] recently found an association between lower levels of vitamin D and SAP. In this study, patients were divided into 3 categories according to their vitamin D status: deficiency (< 25 nmol/L), insufficiency (25–50 nmol/L), and sufficiency (> 50 nmol/L). Patients with vitamin D deficiency had a higher incidence of SAP than patients with vitamin D insufficiency and sufficiency. Moreover, when adjusting by cofounders, vitamin D remained an independent predictor of SAP.

### Acute-phase reactants

C-reactive protein (CRP) has been widely used in clinical practice as an acute-phase marker, which is why it has been also studied deep in the field of SAI. Fluri et al. [49] investigated the role of CRP and other markers in SAI in more than 300 patients. They found that CRP is an independent predictor of SAI, both alone and in combination with clinical factors and other markers. Our group reviewed the studies related to CRP and SAI to date in a systematic review. CRP was observed to be an independent predictor of SAI, and there was an optimal time window between 24 and 48 h after stroke [57].

Serum amyloid A (SAA) is another acute phase-protein that has been associated with the prediction of SAI in a small cohort of patients [58] and was validated as an independent predictor in a recent study with two new cohorts with more than 250 participants each [59]. An association of SAA with SAP was observed in a different study, but in this case, the association was in combination with another marker, the soluble urokinase-type plasminogen activator receptor (suPAR) [60]. When cut-off points were established, the combination of these two markers predicted SAP with high specificity and sensitivity at 48 h after stroke onset. Mid-regional pro-adrenomedullin (MR-proADM) was also associated with SAP but at an earlier time than the SAA/suPAR combination (24 h after stroke).

The relationship of suPAR and MR-proADM with SAI was first explored by Bustamante et al. [61] when evaluating various classical sepsis biomarkers as predictors of SAI. suPAR and MR-proADM were both found to be independent predictors of SAI but in two different cohorts with different time points for blood collection. MR-proADM was found to be an early predictor of SAI in a cohort of 78 patients (based on blood collection at the first 6 h after stroke onset). On the other hand, suPAR was found to be a predictor of SAI in a different cohort, where the time point of the collection was at 24 h after stroke.

Fluri et al. [49] explored procalcitonin (PCT) and copeptin jointly with WBC, and each one by itself was found to be an independent predictor of SAI, SAP, and UTI. Their combination, along with CRP and clinical variables, improved the prediction of infection in stroke patients. Recently, PCT and copeptin were studied as SAP predictors along with other biomarkers by pooling data from two clinical trials (PREDICT [62] and STRAWINSKI [63]). Of all the studied biomarkers, the results showed that only PCT and copeptin were independent predictors of SAP when adjusting for clinical variables such as dysphagia, chronic obstructive pulmonary disease (COPD), hypercholesterolemia, and the National Institute of Health Stroke Scale (NIHSS) at admission [64]. In contrast, at earlier time points, Hu et al. [65] did not find PCT to be useful for the prediction of SAP, only when the clinical suspicion of SAP is already high. In the STRAWINSKI clinical trial, PCT was used as a guide for prophylactic antibiotic therapy. The clinical trial was negative, and PCT was not a predictor of SAI in the study population [63].

### Cytokines and immune-related molecules

Cytokines are important immune mediators in the post-stroke response, so they have been studied as predictors of SAI, including pro-inflammatory IL-6 and anti-inflammatory IL-10. Worthmann et al. [66] studied both, along with lipopolysaccharide-binding protein (LBP) and CRP. The four proteins were measured at different time points in 56 stroke patients, and all of them were associated with SAI incidence at most of the time points, but only CRP and IL-10 were independent predictors of SAI at the earliest time point (6 h after stroke onset). In a different study, IL-6 was an independent predictor of SAI in a cohort of 82 stroke patients and was also associated with mortality [67]. In a meta-analysis, Bustamante et al. [68] reported an independent association of IL-6 with the overcoming of SAI, but the additional predictive value over clinical predictors of SAI was modest.

IL-10 has also been evaluated as a predictor of SAI in other studies. Chamorro et al. [69] reported that IL-10 and circulating monocytes are independent predictors of SAI in 110 stroke patients, and there were higher levels of IL-6 in patients who developed an infection. In line with these results, Ashour et al. [36] reported that IL-10 is an independent predictor of SAI at admission in a study on 60 patients after establishing a cut-off point. Salat et al. [70] explored various cytokines in SAI patients and assessed the role of IL-10, IL-13, and IFN-γ as independent predictors of SAI.

Another study also explored IFN-γ jointly with microRNA-21 (miRNA-21) in a cohort of 54 patients. miRNA-21 was demonstrated to be related to IFN-γ deficiency when it is increased. A significant increase in miRNA-21 levels and a significant decrease in IFN-γ were observed in the peripheral blood of patients who developed SAI, and there was a correlation between both molecules [71]. Another studied cytokine in this field is IL-1 receptor antagonist (IL-1ra). IL1-ra was studied along with IL-10, cortisol, and lymphocyte count, and only IL-1-ra was independently associated with the risk of infection in 112 stroke patients [72].

The expression of human leucocyte antigen-DR (HLA-DR) on peripheral monocytes has also been studied as a predictor of SAI and SAP. In the PREDICT study, HLA-DR was found as an independent predictor of SAP in the presence of dysphagia on day 1 after stroke [62]. Similar results were found by Zhang et al. [73] but focusing on SAI. Even though there was an increase of expression in SAI patients in comparison with non-SAI patients on days 4 and 6 after stroke and not earlier, the authors reported a predictive value of HLA-DR for the risk of infection after a stroke on days 1 and 2.

### Others

The ANS seems to have a role in immunosuppression after stroke, as mentioned in the “Clinical implications (1): the use of cellular and molecular changes as biomarkers for the prediction of stroke-associated infections” section. Heart rate variability (HRV) reflects the activity of this system, so the possible role of HRV in the prediction of SAI was explored by Günther et al. [74]. They found that HRV indices were able to predict the development of SAI before the onset of symptoms.

Suda et al. [75] investigated whether thyroid hormone levels at admission could be associated with the incidence of SAI. They found that lower levels of free triiodothyronine (FT3) were associated with an increased risk of developing SAI. This association remained significant after adjusting by clinical cofounders when establishing a cut-off point for FT3.

### Limitations and future perspectives in biomarkers’ studies

Even though multiple candidates have been studied as possible biomarkers for the prediction of SAI, SAP, or UTI development in stroke patients, as presented in this section, none of them have been proven to have enough predictive value to be used in clinical practice. This could be due to some limitations of these studies. First, only a few studies evaluated the additional predictive value of the biomarkers for the prediction of SAI or SAP. Second, some discrepancies among studies have been observed, perhaps due to the variability between them. The blood collection time might represent one of the most important issues. Usually, patients develop SAP within the first 2–3 days after stroke, so an early detection in the first hours is needed to predict the onset of the infection and to be able to develop therapies to prevent them. Furthermore, most of the biomarker studies have not been evaluated regarding a gold standard test for SAP diagnosis, as there is a lack of a gold standard test for the clinical diagnosis of SAP, and therefore, its diagnosis might differ between studies, hindering its reproducibility. For this reason, it is important to phenotype well all the patients, whether with chest tomography or other tools that could be developed in future studies.

Although none of the presented candidates had enough statistical power by itself to be translated to clinical practice, combining biomarkers with high sensitivity and high specificity could open up new possibilities for researchers. Future studies on SAI and SAP biomarkers should combine the discovery and study of new candidates with the screening of complementary candidates to find a combination with the highest specificity and sensitivity. Hence, more research needs to be done in this field to find a biomarker or combination thereof that could be used to make decisions for the administration of preventive therapies. Multicentric cohorts collecting blood at early time-points and following standardized criteria for pneumonia diagnosis are perhaps the desirable study design.

## Clinical implications (2): moving immunomodulation towards brain recovery and protection against infections

Prophylactic antibiotic therapy does not seem to affect the functional outcomes or the mortality rate in stroke patients [76]. In previous sections, we hypothesized various reasons why antibiotic prophylaxis could not be effective for the prevention of SAP. Another possible explanation for this failure could be that the depletion of part of the gut microbiota by the antibiotics could be favoring the growth of more resistant and aggressive bacterial strains. This, in turn, could be promoting the appearance of pneumonia in stroke patients, not only because of the aspiration of digestive content but also due to the translocation of the gut microbiota to the lung, as has been suggested in experimental studies [77]. This translocation and the subsequent dissemination are mainly due to an impaired intestinal permeability after stroke. Thus, the need for alternative preventive therapies for SAI and SAP is vital, and intense research is being done in this field.

The “Cellular and molecular mechanisms of stroke-induced immunosuppression” section of this review presented the known pathways implicated in SIIS. Along with other clinical factors such as dysphagia, invasive procedures, and comorbidities, SIIS increases the susceptibility to infections in stroke patients [78]. This immunosuppressive response could be attenuated by modulating one or more of the mechanisms of SIIS at the acute phase of stroke, thus reducing the susceptibility of the patients to infection and decreasing the mortality of stroke patients derived from this complication.

In experimental stroke, immunomodulation has been explored as prophylactic therapy for SAI and SAP (Table 1). Several studies have studied the action of the β-adrenoreceptor blockers propranolol and 6-hydroxydopamine (6-OHDA) in experimental rodent models of stroke. They observed that the inhibition of the SNS reduced bacterial infection of the lungs and partially reestablished some of the mechanisms affected by SIIS [18, 19, 33, 79]. The blockade of the HPA axis has also been explored through the administration of the glucocorticoid receptor blocker RU486, which seemed to restore leukocyte counts but did not reduce bacterial lung infections in MCAO rodents [18, 33]. These studies were discussed in detail in the “Cellular and molecular mechanisms of stroke-induced immunosuppression” section.

**Table 1:** Immunomodulating therapies to prevent stroke-associated pneumonia and infections

Mechanism of action	Reference	Drug	Time of administration	Type of study	Major findings
Inhibition of the SNS	Prass et al. 2003 [18]	Propanolol	Immediately before and also 4 and 8 h after MCAO.	Experimental (MCAO mice)	Prevention of lymphocyte apoptosis, lymphopenia, monocytic deactivation and changes in lymphocyte cytokine production; prevention of bacteremia and pneumonia; ↑ survival rates
	Wong et al. 2011 [33]	Propanolol and 6-OHDA	24 h after MCAO	Experimental (MCAO mice)	Reversion of the iNKT cell phenotype induced by MCAO; ↑ survival rates; ↓ bacterial load in blood, lungs, liver, and spleen
	Yan and Zhang 2014 [19]	Propanolol	Immediately before and also 4 and 8 h after MCAO.	Experimental (MCAO mice)	↓ serum levels of MN, NMN and IL-10; ↑ pro-inflammatory cytokines; ↑ spleen volume
	Deng et al. 2016 [79]	6-OHDA	3 days before MCAO	Experimental (MCAO rats)	Reversion of the expression of MHC class II; ↑ TNF-a and IFN-γ levels in LPS-stimulated macrophages in vitro; ↓ NF-κB activation; ↑ β-arrestin2 expression
	Sykora et al 2015 [80]	β1-selective BBs, nonselective BBs	Before and after stroke	Clinical	↓ frequency of pneumonia; association of post-stroke BB treatment with mortality
	Maier et al. 2015 [81]	BBs (mainly metoprolol and bisoprolol)	Before and after stroke	Clinical	No differences in the risk of pneumonia; ↓ mortality.
	Maier et al. 2018 [82]	BBs	Before and after stroke	Clinical	No differences in the rates of pneumonia nor mortality
Inhibition of the HPA axis	Prass et al. 2003 [18]	RU486	24 h, 5 h, and immediately before MCAO	Experimental (MCAO mice)	Prevention of lymphocyte apoptosis, lymphopenia, and monocytic deactivation
Immunomodulation of iNKT cells	Wong et al. 2011 [33]	α-GalCer	24 h after MCAO	Experimental (MCAO mice)	↑ systemic levels of IFN-γ; ↓ stroke-induced neutrophil pulmonary influx and lung edema; ↓ bacterial load in blood, lungs, liver and spleen
Inhibition of CD147	Jin et al. 2019 [83]	CD147 antibody	4 h after MCAO	Experimental (MCAO mice)	↓ lung damage; ↓ lung leukocyte infiltration; ↓ plasma and lung IL-17A
Inhibition of PTEN	Guan et al. 2013 [84]	Bvp	24 h after MCAO	Experimental (MCAO mice)	↓ bacterial loads in lung of bpv-treated mice; restoration of akt activation in the lung; ↓ mortality
GM-CSF	Dames et al. 2018 [85]	Recombinant mGM-CSF	6, 30, and 54 h after MCAO	Experimental (MCAO mice)	↑ leukocyte counts in lung; ↑ WBC count; ↑ long-term outcome

Experimental and clinical studies are represented in this table. In the Major findings column, all the results are referred to the patients or animals treated with the immunomodulator agent in comparison with their respective non-treated controls. *MCAO* middle cerebral artery occlusion, *NA* non-annotated, 6-*OHDA* 6-hydroxydopamine, *iNKT* invariant natural killer T cells, *NM* metanephrine, *NMN* normetanephrine, *IL*-*10* interleukin-10, *MHC* major histocompatibility complex, *TNF*-*α* tumor necrosis factor-α, *IFN*-*γ* interferon-γ, *LBP* lipopolysaccharide binding protein, *BB* beta blocker, α-*GalCer* α-Galactosylceramide, *Bvp* bisperoxovanadium, *GM*-*CSF* granulocyte-macrophage colony-stimulating factor

It is worth mentioning that β-adrenoreceptor antagonists as propranolol seem to be neuroprotective, reducing infarct volumes and improving neurological scores when being administered after MCAO surgery [86]. Thus, β-blockers therapy after stroke could at the same time attenuate brain injury and prevent suffering infections.

However, the action of β-blockers in the reduction of SAI and SAP in humans has been explored in various studies with different degrees of success. In a retrospective study, Sykora et al. [80] reported a reduction in stroke mortality and SAP ratio in stroke patients under β-blocker therapy, along with a reduction of the SAP ratio in patients with pre-stroke β-blocker therapy. However, Maier et al. [81] performed a prospective clinical trial and did not observe differences in the ratio of SAP between patients receiving the post-stroke β-blocker therapy and those that did not. The effect of β-blockers on major stroke patients was also studied, and they did not reduce the incidence of SAP or SAI in this type of patient [82].

Experimental studies have explored other immunomodulation strategies as a preventive therapy for SAP. The inhibition of CD147 seems to reduce the lungs’ susceptibility to bacterial infections in MCAO mice. The protein CD147 is expressed broadly in many leukocyte subtypes and is involved in some immune processes like T cell activation and Th17 cell differentiation. By inhibiting CD147, Jin et al. [83] observed attenuation of lung damage, increased IFN-γ levels in the lungs, and the modulation of leukocyte subpopulation changes in the lungs of MCAO mice. Another immunomodulatory therapy involving the inhibition of PTEN seemed to reduce bacterial lung infections and mortality in MCAO mice, possibly through the restoration of the PI3K/Akt cascade in the lungs [84]. Systemic treatment with the pluripotent cytokine granulocyte-macrophage colony-stimulating factor (GM-CSF) was also explored as an immunomodulating therapy for the prevention of bacterial infections in experimental stroke. This treatment did not reduce the bacterial burden or mortality in MCAO mice, but it did augment antibacterial immune responses and improved the long-term neurological outcome [85].

There has been a lack of success in clinical trials on β-blockers, and there have been discrepancies among them. Furthermore, no other immunomodulation therapy has reached a human-based study phase. Thus, new approaches are needed to find therapeutic targets. Thus far, immunomodulation strategies regarding SAP and SAI prevention have been systemic, with a possible detrimental effect on the brain or other side effects. A local immunomodulation approach could be a new horizon for the discovery of new preventive therapies for SAP.

### The lung as a possible target for new immunomodulatory therapies

One of these new possible targets could be the local lung defenses. The brain-lung crosstalk after acute brain injury is well known, in which the brain and lungs interact closely and bidirectionally. The occurrence of major pulmonary injuries has been observed after acute brain injury. In the case of stroke, patients can suffer other types of pulmonary injuries apart from pneumonia, such as acute lung injury (ALI). An ALI incidence of 22% has been reported in acute stroke patients [87], although the clinical phenotype is mild when compared with other forms of acute brain injury, such as severe traumatic brain injury (TBI) and subarachnoid hemorrhage. Pneumonia, ALI, acute respiratory distress syndrome, and neurogenic pulmonary edema have also been reported after these two conditions [88]. After TBI, a systemic immunosuppression similar to SIIS occurs, which increases the risk of nosocomial infections [89], along with other factors in these neurocritical patients, such as mechanical ventilation. Another reason why the lung could be a target for the local immunomodulation is that its epithelial permeability could be altered by stroke, similarly to the gut epithelium [90].

The mechanisms underlying this interaction between the brain and lungs are still not well understood, although a “double hit model” has been proposed. After an acute brain lesion, the first “hit” involves the secretion of pro-inflammatory cytokines in the brain, the alteration of the BBB permeability, and the activation of the CNS. This causes several systemic alterations that result in local damage to the peripheral organs, such as the lungs, and make them more susceptible to bacterial infections. In turn, the organ damage could exacerbate brain damage [91].

A new approach in the research of preventive therapies for SAP could be derived from the exploration of the cellular and molecular modifications in the lungs after stroke that also contributes to the susceptibility to pneumonia. In a hypothetical scenario, the possible lung damage could be prevented through local immunomodulation, hence decreasing the susceptibility of stroke patients to SAP without hampering neurorestoration in the brain after stroke.

Samary et al. [92] studied this issue in a rat model of focal ischemia. Lung edema and inflammation were observed in ischemic rats, as well as ultrastructural changes in the lung parenchyma. Regarding cytokine levels, they observed increased expressions of TNF-α and IL-6 in the brain and plasma, as well as higher TNF-α levels in the bronchoalveolar lavage fluid (BALF). Furthermore, the phagocytic capability of alveolar macrophages was decreased in stroke animals, along with alterations in different ventilatory parameters, such as the respiratory rate and volume tidal.

In a different study performed in MCAO mice, Austin et al. [93] observed lung inflammation in the animals, but not ALI. They observed BALF inflammation, but they did not observe pulmonary edema or the same pro-inflammatory cytokine expression profile as Samary et al. (2018).

Farris et al. [94] also explored the lung in MCAO mice, and they found alterations in the immune cell niche in the lungs of the ischemic animals, with a decrease of lymphocytes and an increase of alveolar macrophages and neutrophils. The production of multiple chemokines was reduced, including CCL3, CCL20, and CCL22 among others.

These three studies demonstrate the existence of pulmonary alterations after ischemic stroke, but there is some controversy between them. Thus, more research is needed to elucidate the extent of this lung damage and the mechanisms underlying it. In addition, whether the extension and nature of this lung damage is related with the occurrence of lung infections remains to be proved.

## Conclusions

Despite the recent advances in acute stroke treatment with reperfusion therapies, SAI and particularly pneumonia represent some of the major complications after stroke, which have a high impact on patient outcomes and health care systems. Due to the failure of clinical trials on preventive antibiotics, attractive pathways for researchers and clinicians against this complication involve the prediction and early detection with biomarkers and preventive interventions with immunomodulators. However, the rapidly changing clinical scenario of stroke patients creates new challenges for such research.

Despite the undoubted clinical benefits, the generalization of mechanical thrombectomy exposes hyperacute stroke patients to situations of high risk for aspiration, such as long transfers and prolonged interventions. Therefore, the need to move to very early interventions for both risk stratification and preventive measures points to the identification of earlier signals of the impaired immune response to identify both biomarkers and therapeutic targets. From a therapeutic point of view, researchers also have to consider the potential side-effects on the brain of enhancing systemic immunity. In this sense, the lungs arise as a potential therapeutic target to be explored in the next years, particularly the lung damage caused by brain ischemia, as well as the mechanisms and pathways that are involved.

## Supplementary Information

**Additional file 1: Table S1.** Studied biomarkers for the prediction of SAI, SAP or UTI. This table summarizes the biomarkers presented in section 4 alphabetically ordered.

## Data Availability

Not applicable.

## Abbreviations

6-OHDA: 6-Hydroxydopamine
ALI: Acute lung injury
ANS: Autonomic nervous system
ArgI: Arginase-1
ARRB2: β-arrestin2
BALF: Bronchoalveolar lavage fluid
BBB: Blood-brain barrier
CD: Cluster of differentiation
CNS: Central nervous system
COPD: Chronic obstructive pulmonary disease
CRP: C-reactive protein
DAMPs: Damage-associated molecular pattern
DHT: Dihydrotestosterone
FT3: Free triiodothyronine
GM-CSF: Granulocyte-macrophage colony-stimulating factor
HDL: High-density lipoprotein
HLA-DR: Human leucocyte antigen-DR
HMGB1: High mobility group box 1
HPA: Hypothalamic-pituitary-adrenal
HRV: Heart rate variability
IFN-γ: Interferon-γ
IL: Interleukin
iNKT: Hepatic invariant natural killer T
MCAO: Middle cerebral artery occlusion
MHR: Monocyte to HDL cholesterol ratio
miRNA-21: MicroRNA-21
MN: Metanephrine
MR-proADM: Mid-regional pro-adrenomedullin
NF-κB: Nuclear factor kappa B
NIHSS: National Institute of Health Stroke Scale
NLR: Neutrophil-to-lymphocyte ratio
NMN: Normetanephrine
PCT: Procalcitonin
PISCES: Pnemonia In Stroke Consensus
PNS: Parasympathetic nervous system
RAGE: Receptor of advanced glycation end-products
SAA: Serum amyloid A
SAI: Stroke-associated infection
SAP: Stroke-associated pneumonia
SIIS: Stroke-induced immunosuppression
SNS: Sympathetic nervous system
suPAR: Soluble urokinase-type plasminogen activator receptor
TBI: Traumatic brain injury
Th: T-helper
THRCT: Thorax high resolution computed tomography
TNF-α: Tumor necrosis factor-α
TRAF4: Tumor necrosis factor receptor-associated 4
UTI: Urinary tract infection
WBC: White blood cell
α7nAChR: α7 nicotinic acetylcholine receptor
