# Supplementary material for: Stroke-induced immunosuppression: implications for the prevention and prediction of post-stroke infections
Source: J Neuroinflammation. 2021 Jun 6;18:127. doi: 10.1186/s12974-021-02177-0 (PMC8183083; doi:10.1186/s12974-021-02177-0)
Supplement: Supplementary file 1 — Additional file 1: Table S1. Studied biomarkers for the prediction of SAI, SAP or UTI. This table summarizes the biomarkers presented in section 4 alphabetically ordered. [file 12974_2021_2177_MOESM1_ESM.docx]

**Table S1. Studied biomarkers for the prediction of SAI, SAP or UTI.**

| **Marker** | **References** | **Outcome** | **Number of patients** | **Diagnostic criteria** | **Sample collection time (from stroke onset)** | **Direction** | **Clinical findings** |
| --- | --- | --- | --- | --- | --- | --- | --- |
| **Albumin** | Dziedzic et al., 2006 [1] | SAP | 705 | Own criteria | First 36h | ↓ | Independent predictor of SAP |
| **Blood glucose** | Zonneveld et al., 2017 [2] | SAI, SAP and UTI | 1676 | CDC criteria [3] | At admission | ↑ | Independent predictor of SAI, SAP and UTI |
| **Copeptin** | Fluri et al., 2012 [4] | SAI, SAP and UTI | 383 | CDC criteria | First 72h | ↑ | Independent predictor of SAI, SAP and UTI |
|  | Hotter et al., 2020 [5] | SAP | 573 | PISCES criteria [6] | First 40h | ↑ | Independent predictor of SAP |
| **CRP** | Fluri et al., 2012 [4] | SAI, SAP and UTI | 383 | CDC criteria | First 72h | ↑ | Independent predictor of SAI, SAP and UTI |
|  | Bustamante et al., 2017b [7] | SAI | 699 (systematic review) | Depending on the study | First 24h, 24-48h and 48-72h | ↑ | Independent predictor of SAI between 24 and 48h. |
| **FT3** | Suda et al., 2018b [8] | SAI | 520 | CDC criteria | At admission | ↓ | Independent predictor of SAI |
| **HDL** | Rodríguez-Sanz et al., 2013 [9] | SAI | 1385 | Own criteria | Morning following admission | ↑ | Independent predictor of lower risk of SAI |
| **Heart rate variability** | Günther et al., 2012 [10] | SAI | 43 | CDC criteria | First 48h and 4±1 days | NA | Independent predictor of SAI |
| **HLA-DR** | Zhang et al., 2009 [11] | SAI | 53 | Own criteria | 1, 2, 4, 6 and 14 days after stroke | ↓ | Predictive value for SAI at days 1 and 2 |
|  | Hoffmann et al., 2017 [12] | SAP | 479 | CDC criteria |  | ↓ |  |
| **IFN-γ** | Salat et al., 2013 [13] | SAI | 92 | CDC criteria | After the administration of thrombolytic therapy | ↓ | Independent predictor of SAI |
|  | Lin et al., 2016 [14] | SAI | 54 | ESPIAS clinical trial criteria [15] | First 24h | ↓ | Lower levels in patients with SAI |
| **IL1-ra** | Tanzi et al., 2011 [16] | SAI | 112 | Own criteria | 1, 3, 7, 30 and 90 days | ↑ | Independent predictor of SAI at 72h |
| **IL-6** | Worthmann et al., 2015 [17] | SAI | 56 | Local clinical practice criteria | 6h, 12h, 24h, 3 days and 7 days | ↑ | Higher levels in SAI patients at 12h, 24h, day 3 and day 7 |
|  | Kwan et al., 2013 [18] | SAI | 82 | Langhorne et al., 2000 | First 72h | ↑ | Independent predictor of SAI |
|  | Chamorro et al., 2006 [20] | SAI | 110 | ESPIAS clinical trial criteria | Admission, 1, 2, 3, 4 and 7 days | ↑ | Higher levels in patients with SAI at days 1, 2 and 3 |
| **IL-10** | Worthmann et al., 2015 [17] | SAI | 56 | Local clinical practice criteria | 6h, 12h, 24h, 3 days and 7 days | ↑ | Independent predictor of SAI at 6h |
|  | Chamorro et al., 2006 [20] | SAI | 110 | ESPIAS clinical trial criteria | Admission, 1, 2, 3, 4 and 7 days | ↑ | Independent predictor of SAI at admission |
|  | Ashour et al., 2016 [21] | SAI | 60 | Engel et al., 2010; Stott et al., 2009 | Admission, 4 and 7 days | ↑ | Independent predictor of SAI at admission |
|  | Salat et al., 2013 [13] | SAI | 92 | CDC criteria | After the administration of thrombolytic therapy | ↑ | Independent predictor of SAI |
| **IL-13** | Salat et al., 2013 [13] | SAI | 92 | CDC criteria | After the administration of thrombolytic therapy | ↑ | Independent predictor of SAI |
| **LBP** | Worthmann et al., 2015 [17] | SAI | 56 | Local clinical practice criteria | 6h, 12h, 24h, 3 days and 7 days | ↑ | Higher levels in SAI patients at 12h, 24h, day 3 and day 7 |
| **MHR** | Sun et al., 2020 [24] | SAP | 803 | PISCES criteria | First 24h | ↑ | Independent predictor of SAP (highest quartile) |
| **miRNA-21** | Lin et al., 2016 [14] | SAI | 54 | ESPIAS clinical trial criteria | First 24h | ↑ | Higher levels in patients with SAI |
| **Monocyte count** | Sun et al., 2020 [24] | SAP | 803 | PISCES criteria | First 24h | ↑ | Independent predictor of SAP (highest quartile) |
| **MR-proADM** | Zapata-Arriaza et al., 2019 [25] | SAP | 45 | THRCT [26] | First 24h, 1 and 2 days | ↑ | Associated with SAP at 24h |
|  | Bustamante et al., 2017a [27] | SAI | 125 | CDC criteria | Baseline (6h) and 24 hours (two different groups) | ↑ | Independent predictor of SAI at baseline |
| **NK cells count** | De Raedt et al., 2015 [28] | SAI | 59 | CDC criteria | At admission, 1, 3, 5 and 7 days | ↑ | Higher levels in patients with SAI at admission |
| **NLR** | Nam et al., 2018 [29] | SAP | 1317 | PISCES criteria | First 24h | ↑ | Higher levels in patients with SAP |
| **PCT** | Fluri et al., 2012 [4] | SAI, SAP and UTI | 383 | CDC criteria | First 72h | ↑ | Independent predictor of SAI, SAP and UTI |
|  | Hotter et al., 2020 [5] | SAP | 573 | PISCES criteria | First 40h | ↑ | Independent predictor of SAP |
|  | Hug et al., 2011 [30] | SAP | 50 | Own criteria | Day 1 and 4 | ↑ | No predictive value for SAP |
| **Prealbumin** | Ye et al., 2017 [31] | SAI | 104 | Own criteria | First day of admission | ↓ | Lower levels in patients with SAI |
| **SAA** | Azurmendi et al., 2017 [32] | SAI | 40 | CDC criteria | First 72h, 1, 3 and 5 days | ↑ | Associated with SAI at all time points |
|  | Zapata-Arriaza et al., 2019 [25] | SAP | 45 | THRCT | First 24h, 1 and 2 days | ↑ | Associated with SAP in combination with suPAR at day 2 |
|  | Schweizer et al., 2020 [33] | SAI | 283 (cohort A) and 367 (cohort B) | CDC criteria | First 72h (cohort A) and first 24h (cohort B) | ↑ | Independent predictor of SAI in both cohorts. |
| **suPAR** | Zapata-Arriaza et al., 2019 [25] | SAP | 45 | THRCT | First 24h, 1 and 2 days | ↑ | Associated with SAP in combination with SAA at day 2 |
|  | Bustamante et al., 2017a [27] | SAI | 125 | CDC criteria | Baseline (6h) and 24 hours (two different groups) | ↑ | Independent predictor of SAI at 24h |
| **Vitamin D** | Huang et al., 2019 [34] | SAP | 863 | CDC criteria | First day of admission | ↓ | Independent predictor of SAP |
| **WBC count** | Fluri et al., 2012 [4] | SAI, SAP and UTI | 383 | CDC criteria | First 72h | ↑ | Independent predictor of SAI, SAP and UTI |

This table summarizes the biomarkers presented in section 4 alphabetically ordered. The table includes the reference of the publication in which each biomarker was studied, outcome of the biomarker, number of patients in the study, diagnostic criteria for infections, time of blood sample collection after stroke, direction of the expression alteration (upregulated ↑ or downregulated ↓) regarding the control group, and the clinical finding. The references can be found in the main text. Abbreviations: WBC: white blood cell, NLR: neutrophil-to-lymphocyte ratio, NK: natural killer, HDL: high density lipoprotein, NA: non-annotated, CRP: C-reactive protein, SAA: serum amyloid A, suPAR: soluble urokinase receptor, MR-proADM: mid-regional proadrenomedullin, PCR: procalcitonin, IL: interleukin, IFN-γ: interferon-γ, LBP: lipopolysaccharide binding protein, MHR: Monocyte to high-density lipoprotein cholesterol ratio, miRNA-21: microRNA-21, HLA-DR: Human Leukocyte Antigen – DR, FT3: free triiodothyronine, CDC: Centers for Disease Control and Prevention, THRCT: Thorax high-resolution computed tomography, SAI: Stroke-associated infection, SAP: Stroke-associated pneumonia, UTI: Urinary tract infection, PISCES: Pneumonia in stroke consensus.

**BIBLIOGRAPHY**

1. Dziedzic T, Pera J, Klimkowicz A, Turaj W, Slowik A, Rog TM, et al. Serum albumin level and nosocomial pneumonia in stroke patients. Eur J Neurol. 2006;13:299–301.

2. Zonneveld TP, Nederkoorn PJ, Westendorp WF, Brouwer MC, Van De Beek Di, Kruyt ND. Hyperglycemia predicts poststroke infections in acute ischemic stroke. Neurology. 2017;88:1415–21.

3. Horan TC, Andrus M, Dudeck MA. CDC/NHSN surveillance definition of health care-associated infection and criteria for specific types of infections in the acute care setting. Am J Infect Control. 2008;36:309–32.

4. Fluri F, Morgenthaler NG, Mueller B, Christ-Crain M, Katan M. Copeptin, Procalcitonin and Routine Inflammatory Markers-Predictors of Infection after Stroke. PLoS One. 2012;7.

5. Hotter B, Hoffmann S, Ulm L, Montaner J, Bustamante A, Meisel C, et al. Inflammatory and stress markers predicting pneumonia, outcome, and etiology in patients with stroke: Biomarkers for predicting pneumonia, functional outcome, and death after stroke. Neurol Neuroimmunol neuroinflammation. 2020;7.

6. Smith CJ, Kishore AK, Vail A, Chamorro A, Garau J, Hopkins SJ, et al. Diagnosis of Stroke-Associated Pneumonia: Recommendations From the Pneumonia in Stroke Consensus Group. Stroke. 2015;46:2335–40.

7. Bustamante A, Vilar-Bergua A, Guettier S, Sánchez-Poblet J, García-Berrocoso T, Giralt D, et al. C-Reactive Protein in the Detection of Post-Stroke Infections: Systematic Review and Individual Participant Data Analysis. J Neurochem. 2017;141:305–14.

8. Suda S, Aoki J, Shimoyama T, Suzuki K, Sakamoto Y, Katano T, et al. Low Free Triiodothyronine at Admission Predicts Poststroke Infection. J Stroke Cerebrovasc Dis [Internet]. Elsevier Inc.; 2018;27:397–403. Available from: https://doi.org/10.1016/j.jstrokecerebrovasdis.2017.09.012

9. Rodríguez-Sanz A, Fuentes B, Martínez-Sánchez P, Prefasi D, Martínez-Martínez M, Correas E, et al. High-density lipoprotein: A novel marker for risk of in-hospital infection in acute ischemic stroke patients? Cerebrovasc Dis. 2013;35:291–7.

10. Günther A, Salzmann I, Nowack S, Schwab M, Surber R, Hoyer H, et al. Heart rate variability - a potential early marker of sub-acute post-stroke infections. Acta Neurol Scand. 2012;126:189–96.

11. Zhang DP, Yan FL, Xu HQ, Zhu YX, Yin Y, Lu HQ. A decrease of human leucocyte antigen-DR expression on monocytes in peripheral blood predicts stroke-associated infection in critically-ill patients with acute stroke. Eur J Neurol. 2009;16:498–505.

12. Hoffmann S, Harms H, Ulm L, Nabavi DG, Mackert BM, Schmehl I, et al. Stroke-induced immunodepression and dysphagia independently predict stroke-associated pneumonia – The PREDICT study. J Cereb Blood Flow Metab. 2017;37:3671–82.

13. Salat D, Penalba A, García-Berrocoso T, Campos-Martorell M, Flores A, Pagola J, et al. Immunological biomarkers improve the accuracy of clinical risk models of infection in the acute phase of ischemic stroke. Cerebrovasc Dis. 2013;35:220–7.

14. Lin SP, Ye S, Chen XH, Jiang HL, Mao HF, Chen MT, et al. Increased expression of microRNA-21 in peripheral blood mediates the down-regulation of IFN-γ and increases the prevalence of stroke-associated infection. J Neurol Sci [Internet]. Elsevier B.V.; 2016;366:235–9. Available from: http://dx.doi.org/10.1016/j.jns.2016.03.034

15. Chamorro A, Horcajada JP, Obach V, Vargas M, Revilla M, Torres F, et al. The Early Systemic Prophylaxis of Infection After Stroke study: a randomized clinical trial. Stroke. United States; 2005;36:1495–500.

16. Tanzi P, Cain K, Kalil A, Zierath D, Savos A, Gee JM, et al. Post-stroke infection: A role for IL-1ra? Neurocrit Care. 2011;14:244–52.

17. Worthmann H, Tryc AB, Dirks M, Schuppner R, Brand K, Klawonn F, et al. Lipopolysaccharide binding protein, interleukin-10, interleukin-6 and C-reactive protein blood levels in acute ischemic stroke patients with post-stroke infection. J Neuroinflammation. 2015;12:1–9.

18. Kwan J, Horsfield G, Bryant T, Gawne-Cain M, Durward G, Byrne CD, et al. IL-6 is a predictive biomarker for stroke associated infection and future mortality in the elderly after an ischemic stroke. Exp Gerontol. 2013;48:960–5.

19. Langhorne P, Stott D, Robertson L, MacDonald J, Jones L, McAlpine C, et al. Medical complications after stroke. Stroke. 2000;31:1223–9.

20. Chamorro Á, Amaro S, Vargas M, Obach V, Cervera Á, Torres F, et al. Interleukin 10, monocytes and increased risk of early infection in ischaemic stroke. J Neurol Neurosurg Psychiatry. 2006;77:1279–81.

21. Ashour W, Al-Anwar AD, Kamel AE, Aidaros MA. Predictors of early infection in cerebral ischemic stroke. J Med Life. 2016;9:163–9.

22. Engel O, Dirnagl U, Meisel A. Infection - An amendment to the stroke model guidelines. J Exp Stroke Transl Med. 2010;3:29–32.

23. Stott DJ, Falconer A, Miller H, Tilston JC, Langhorne P. Urinary tract infection after stroke. QJM. 2009;102:243–9.

24. Sun Y, Lu J, Zheng D, Qian J, Zhang H, Xing D, et al. Predictive value of monocyte to HDL cholesterol ratio for stroke-associated pneumonia in patients with acute ischemic stroke. Acta Neurol Belg [Internet]. Springer International Publishing; 2020; Available from: https://doi.org/10.1007/s13760-020-01418-y

25. Zapata-Arriaza E, Mancha F, Bustamante A, Moniche F, Pardo-Galiana B, Serrano-Gotarredona P, et al. Biomarkers predictive value for early diagnosis of Stroke-Associated Pneumonia. Ann Clin Transl Neurol. 2019;6:1882–7.

26. Zapata-Arriaza E, Serrano-Gotarredona P, Navarro-Herrero S, Moniche F, Pardo-Galiana B, Pallisa E, et al. Chest computed tomography findings and validation of clinical criteria of stroke associated pneumonia. J Stroke. 2019;21:217–9.

27. Bustamante A, García-Berrocoso T, Penalba A, Giralt D, Simats A, Muchada M, et al. Sepsis biomarkers reprofiling to predict stroke-associated infections. J Neuroimmunol [Internet]. Elsevier B.V; 2017;312:19–23. Available from: http://dx.doi.org/10.1016/j.jneuroim.2017.08.012

28. De Raedt S, De Vos A, Van Binst AM, De Waele M, Coomans D, Buyl R, et al. High natural killer cell number might identify stroke patients at risk of developing infections. Neurol Neuroimmunol NeuroInflammation. 2015;2:e71.

29. Nam KW, Kim TJ, Lee JS, Kwon HM, Lee YS, Ko SB, et al. High neutrophil-to-lymphocyte ratio predicts stroke-associated pneumonia. Stroke. 2018;49:1886–92.

30. Hug A, Mürle B, Dalpke A, Zorn M, Liesz A, Veltkamp R. Usefulness of serum procalcitonin levels for the early diagnosis of stroke-associated respiratory tract infections. Neurocrit Care. 2011;14:416–22.

31. Ye S, Lin SP, Wu K, Fan Y, Xu M. Serum prealbumin is a predictive biomarker for stroke-associated infection after an ischemic stroke. Int J Neurosci. 2017;127:601–5.

32. Azurmendi L, Lapierre-Fetaud V, Schneider J, Montaner J, Katan M, Sanchez JC. Proteomic discovery and verification of serum amyloid A as a predictor marker of patients at risk of post-stroke infection: A pilot study. Clin Proteomics. 2017;14–27.

33. Schweizer J, Bustamante A, Lapierre-Fétaud V, Faura J, Scherrer N, Azurmendi Gil L, et al. SAA (Serum Amyloid A): A Novel Predictor of Stroke-Associated Infections. Stroke. 2020;51:3523–30.

34. Huang GQ, Cheng HR, Wu YM, Cheng QQ, Wang YM, Fu JL, et al. Reduced vitamin D levels are associated with stroke-associated pneumonia in patients with acute ischemic stroke. Clin Interv Aging. 2019;14:2305–14.
